# Annexin A1 Tethers Membrane Contact Sites that Mediate ER to Endosome Cholesterol Transport

**DOI:** 10.1016/j.devcel.2016.05.005

**Published:** 2016-06-06

**Authors:** Emily R. Eden, Elena Sanchez-Heras, Anna Tsapara, Andrzej Sobota, Tim P. Levine, Clare E. Futter

**Affiliations:** 1 UCL Institute of Ophthalmology, London EC1V 9EL, UK; 2 Technical University of Crete, 73100 Chania, Greece; 3 Nencki Institute of Experimental Biology, 02-093 Warsaw, Poland

## Abstract

Membrane contact sites between the ER and multivesicular endosomes/bodies (MVBs) play important roles in endosome positioning and fission and in neurite outgrowth. ER-MVB contacts additionally function in epidermal growth factor receptor (EGFR) tyrosine kinase downregulation by providing sites where the ER-localized phosphatase, PTP1B, interacts with endocytosed EGFR before the receptor is sorted onto intraluminal vesicles (ILVs). Here we show that these contacts are tethered by annexin A1 and its Ca^2+^-dependent ligand, S100A11, and form a subpopulation of differentially regulated contact sites between the ER and endocytic organelles. Annexin A1-regulated contacts function in the transfer of ER-derived cholesterol to the MVB when low-density lipoprotein-cholesterol in endosomes is low. This sterol traffic depends on interaction between ER-localized VAP and endosomal oxysterol-binding protein ORP1L, and is required for the formation of ILVs within the MVB and thus for the spatial regulation of EGFR signaling.

## Highlights

• Multiple biochemically distinct populations of ER-endocytic organelle contact sites
• Annexin A1-S100A11 interaction tethers ER contacts with EGFR-positive endosomes
• ER-to-endosome cholesterol transfer to support ILV formation requires contact sites
• ER to endosome cholesterol transport depends on direct VAPA-ORP1L interaction

## Teaser

Eden et al. identify annexin A1 as a tether for ER membrane contact sites with EGFR-containing endosomes. Under low cholesterol conditions, annexin A1-regulated contact sites are required for ER-derived cholesterol transport to endosomes. This is necessary for the formation of intraluminal vesicles within the endosomal lumen that spatially regulate EGFR signaling.

## Introduction

The ER forms an extensive network of membrane contact sites (MCSs), microdomains of close membrane apposition (<30 nm), with a diverse range of functionally distinct organelles, providing an important means of non-vesicular communication between organelles. Although only recently described (Eden et al., 2010, Rocha et al., 2009), MCSs between the ER and the endocytic pathway are extremely abundant (Friedman et al., 2013, Kilpatrick et al., 2013), suggesting important physiological roles (Raiborg et al., 2015b). Indeed functions in endosomal positioning (Rocha et al., 2009) and defining the timing and position of endosome fission during cargo sorting (Rowland et al., 2014) have been reported. ER-endosome MCSs were also recently found to mediate endosome translocation to and fusion with the plasma membrane, promoting protrusion and neurite outgrowth (Raiborg et al., 2015a). MCSs provide sites of interaction for the ER-localized phosphatase, PTP1B, with endocytosed epidermal growth factor receptor (EGFR) and components of the endosomal sorting complex required for transport (ESCRT) machinery (Eden et al., 2010, Stuible et al., 2010). PTP1B activity dampens EGFR signaling, not only by dephosphorylating the EGFR, but also by promoting EGF-stimulated intraluminal vesicle (ILV) formation (Eden et al., 2010), a process that sequesters the catalytic domain of the receptor from cytoplasmic substrates prior to lysosomal degradation.

The molecular composition of ER contacts with the endocytic pathway remains poorly understood, hampering functional studies. MCSs are stabilized by tethering complexes that maintain close proximity between apposing membranes. Vesicle-associated membrane protein-associated proteins (VAPs) are conserved ER membrane proteins that recruit binding partners to multiple MCSs between the ER and other organelles (Prinz, 2014) by binding FFAT motifs, which are predominantly found in lipid transfer proteins (Loewen and Levine, 2005). Two sterol-binding proteins, ORP1L (Rocha et al., 2009) and STARD3 (Alpy et al., 2013), that both contain FFAT motifs, interact with VAP at MCSs between the ER and endosomes. ORP1L is recruited to Rab7-positive late endosomes, distinct from the earlier endosomes that stain for STARD3 (van der Kant et al., 2013), while both early and late EGFR-containing multivesicular endosomes/bodies (MVBs) can form MCSs with the ER (Eden et al., 2010), together suggesting the existence of multiple populations of MCS between the ER and endocytic organelles. We previously showed that EGFR traffics in a subpopulation of MVBs in which annexin A1 promotes ILV formation by an unknown mechanism (White et al., 2006). Annexin A1 is a substrate of EGFR tyrosine kinase (Gerke and Moss, 2002) and can mediate membrane aggregation in vitro (Blackwood and Ernst, 1990) and so is itself a candidate tether. We hypothesized that annexin A1's primary role at the MVB could be in MCS formation, which in turn is required for ILV formation. MCSs likely facilitate ILV formation by allowing PTP1B interaction with endosomal ESCRT proteins (Eden et al., 2010, Stuible et al., 2010). Here we demonstrate the presence of multiple biochemically distinct MCSs between the ER and endocytic organelles. Annexin A1 is a key regulator of both ER contacts with EGFR-positive MVBs and EGF-stimulated ILV formation, a process that we find requires cholesterol. When there is not enough cholesterol in the endocytic pathway, annexin A1-regulated MCSs are required for ORP1L/VAP-dependent transport of ER-derived cholesterol to MVBs to support ILV formation.

## Results

### Annexin A1 Tethers a Subpopulation of Differentially Regulated MCSs between the ER and Endocytic Organelles that Provide Sites for PTP1B-EGFR Interaction

**Figure 1:**
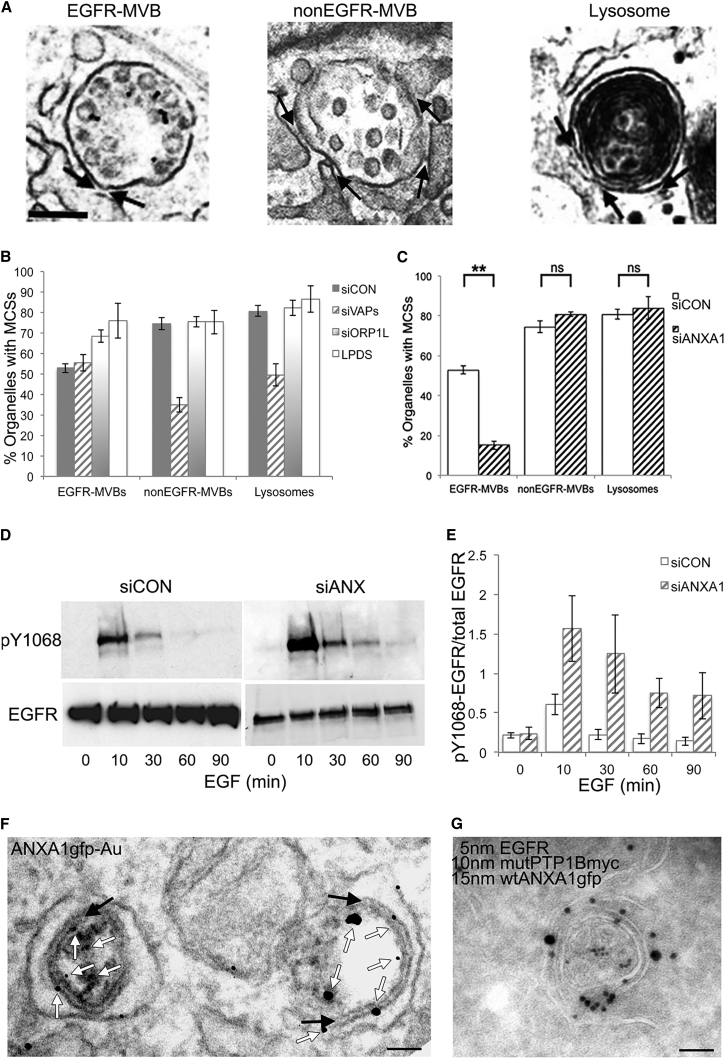
Role of VAPs, ORP1L, and Annexin A1 in MCSs between the ER and Different Endocytic Organelles (A and B) HeLa cells transfected with non-targeting siRNA (siCON) or siRNA targeting VAPA and VAPB (siVAPs) or ORP1L (siORP1L) or cultured overnight in LPDS were stimulated with EGF and anti-EGFR gold (30 min). ER MCSs (arrows) with endocytic organelles (examples in A) were quantified (B). Data are mean ± SD of three experiments. Scale bar, 200 nm. (C) As (B), with siRNA targeting annexin A1 (siANXA1). ns, not significant p > 0.05; ^∗∗^p < 0.01. (D and E) HeLa cells transfected with non-targeting siRNA (siCON) or siRNA targeting annexin A1 (siANXA1) were stimulated with EGF as indicated, cell lysates blotted with antibodies to phosphotyrosine(pY)1068 or total EGFR (D), and quantified (E). Data are mean ± SD of three experiments. (F) HeLa cells transfected with WT annexin A1-gfp and mutant (D181A) PTP1B-myc were stimulated with EGF-HRP (60 min) and stained for GFP using pre-embedding labeling. GFP (white arrows) is visible at MCSs (black arrows) between the ER and EGFR-MVBs containing electron-dense EGF-HRP DAB reaction product. Scale bar, 200 nm. (G) HeLa cells transfected with annexin A1-gfp and D181APTP1B-myc were stimulated with EGF and anti-EGFR-5 nm gold (30 min). Cryosections were labeled with anti-myc (10 nm gold) and anti-gfp (15 nm gold). Scale bar, 200 nm. See also Figure S1.

We have used electron microscopy (EM) to unequivocally identify MCSs, while also allowing the distinction between MVBs (containing discrete ILVs) and electron-dense lysosomes (Figure 1A). Co-incubating EGF-stimulated cells with an antibody to the EGFR extracellular domain coupled to gold allows EGFR-containing and non-EGFR-containing MVB subpopulations to be distinguished (Figure 1A). Although MCSs with a given MVB may not be in the plane of a random section, we found MCS quantification in random sections to be comparable with that achieved by serial sectioning (Figure S1A). Focusing first on the potential role of the VAP-ORP1L interaction, we found that ER contacts with EGFR-MVBs were unaffected by VAP depletion, but MCSs with non-EGFR-MVBs and lysosomes were reduced by approximately 50% (Figures 1B and S1B). This shows that more than one population of MCSs exists between the ER and endocytic organelles. Consistent with a role for VAPs in tethering MCSs between the ER and both non-EGFR-MVBs and lysosomes, we found that cfp-VAPA localized to the ER including sites of contact with both these organelles (Figure S1C). Surprisingly, MCS formation was not impaired in cells depleted of ORP1L, but MCSs with EGFR-MVBs were increased (Figures 1B and S1B). As previously reported (Rocha et al., 2009), ER-endosome MCSs were increased when cells were cultured in lipoprotein-deficient serum (LPDS), conditions which favor VAP-ORP1L interaction. However, this effect was confined to the subpopulation of MCSs with EGFR-MVB (Figure 1B), although the already high percentage of non-EGFR-MVBs and lysosomes with an MCS might render further potential increases difficult to discern. Nevertheless, our data suggest that EGFR-MVBs have a requirement for cholesterol that is revealed when cells are deprived of exogenous lipoprotein.

In contrast to the effects of VAP depletion, when we depleted cells of annexin A1, MCSs between the ER and EGFR-MVBs were considerably reduced (Figures 1C and S1B). ER contacts with both non-EGFR-MVBs and lysosomes were unaffected, confirming the existence of multiple biochemically distinct populations of ER-endocytic organelle MCSs. The loss of ER contacts with EGFR-MVBs on annexin A1 depletion was accompanied by increased and prolonged EGFR tyrosine phosphorylation (Figures 1D and 1E). This was not observed in cells depleted of ORP1L or VAPs (Figures S1D and S1E). Moreover, the accelerated EGFR tyrosine dephosphorylation observed in cells stably overexpressing wild-type (WT) PTP1BMyc (Eden et al., 2010) was lost on depletion of annexin A1 (Figure S1F), suggesting that annexin A1-regulated MCSs provide sites for PTP1B-mediated EGFR dephosphorylation. We next determined whether annexin A1 co-localizes with PTP1B at MCSs, taking advantage of our previous demonstration that expression of a substrate-trapping (D181A) mutant PTP1B causes the formation of extended contacts between the ER and EGFR-MVBs (Eden et al., 2010). Localization of annexin A1-gfp by pre-embedding labeling, identifying PTP1B-containing MCSs by virtue of their size (Figure 1F), or by cryo-immunoEM (Figure 1G), showed that annexin A1 was present at MCSs between the ER and EGFR-MVBs. Annexin A1 also localized at and proximal to the point of contact between the ER and MVBs in the absence of mutant PTP1B expression (Figure S1G). These data indicate a direct role for annexin A1 in tethering the two membranes at MCSs required for PTP1B-mediated EGFR dephosphorylation.

### Correlation between Annexin A1-Regulated MCS Formation and EGF-Stimulated ILV Formation

**Figure 2:**
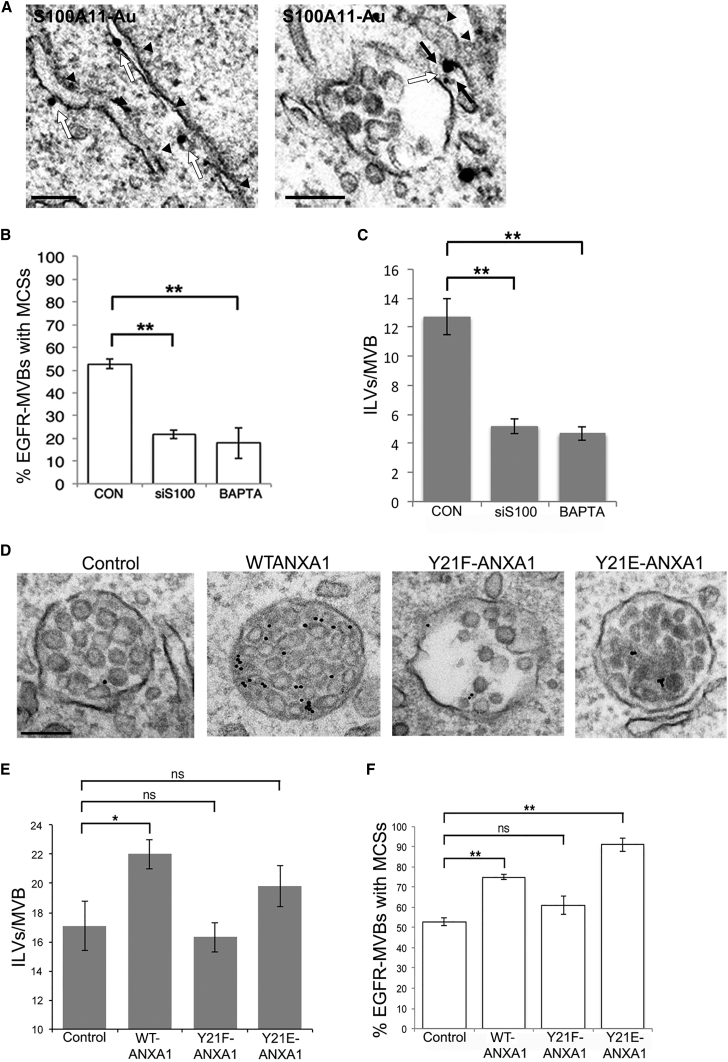
Correlation between Requirements for ER MCSs with EGFR-MVBs and ILV Formation (A) HeLa cells were stained for endogenous S100A11 using pre-embedding labeling. Staining (white arrows) is visible on the ER (arrowheads) and at MCSs (black arrows) between the ER and MVBs. Scale bar, 200 nm. (B and C) HeLa cells transfected with non-targeting siRNA (CON) and siRNA targeting S100A11 (siS100) or incubated with BAPTA were stimulated with EGF and anti-EGFR gold and MCSs with EGFR-MVBs (B) and ILVs/EGFR-MVB (C) quantified. Data are means ± SD of three experiments. ^∗∗^p < 0.01. (D–F) HeLa cells mock transfected (Control) or transfected with WT annexin A1 or annexin A1 mutants were stimulated and quantified as above. Data are means ± SD of three experiments. ns, not significant p > 0.05; ^∗^p < 0.05, ^∗∗^p < 0.01. See also Figure S2.

We previously demonstrated that annexin A1 is required for EGF-stimulated ILV formation (White et al., 2006), but the mechanism whereby it promotes this ESCRT-dependent process is unclear. Although manipulating PTP1B expression dramatically affects ILV formation, it has only small effects on MCS formation (Eden et al., 2010), suggesting that PTP1B's role in ILV formation is through modulating endosomal ESCRT or EGFR phosphorylation. However, the marked reduction in MCS formation upon annexin A1 depletion suggests that annexin A1's main role in ILV formation could be through MCS formation. In this case, modulating annexin A1 activity should affect both MCS and ILV formation. Annexin A1 can form a Ca^2+^-dependent heterotetramer with S100A11 (Rety et al., 2000) so we determined the intracellular distribution of S100A11. Its predominant ER localization, including at MCSs between the ER and MVBs (Figure 2A), raised the possibility that its interaction with endosomal annexin A1 might bridge the two membranes at the contact. Consistently, depletion of S100A11 (Figure S2A) or treatment with the cell-permeable calcium chelator, BAPTA-AM, severely disrupted ER contacts with EGFR-MVBs (Figure 2B). Furthermore, the same treatments severely disrupted EGF-stimulated ILV formation (Figure 2C), revealing a striking correlation between MCS and ILV formation. S100A11 depletion also resulted in increased and prolonged EGFR tyrosine phosphorylation (Figures S2B and S2C), consistent with a role for annexin A1/S100A11 interactions in tethering MCSs that provide sites for PTP1B-mediated effects at the endosome.

We previously showed that EGF-stimulated ILV formation could be restored in annexin A1 knockout cells by overexpression of WT but not a tyrosine phosphorylation mutant of annexin A1 (Y21F) (White et al., 2006). Therefore, to further probe the requirements for MCS and ILV formation, we expressed phosphorylation mutant annexin A1 constructs (Figure S2D). Overexpression of WT annexin A1 increased ILVs/EGFR-MVB, as did phosphomimetic Y21E-annexin A1, although this did not reach statistical significance. Expression of Y21F-annexin A1 (that cannot be tyrosine phosphorylated) had no effect on ILV formation (Figures 2D and 2E). Importantly, WT and phosphomimetic, but not phosphorylation mutant annexin A1, also increased ER MCSs with EGFR-MVBs (Figures 2D and 2F), correlating with the effects of the same constructs on ILV formation (Figure 2E). This suggests that EGF-stimulated annexin A1 phosphorylation is necessary for its role in MCS formation. In addition, we found an interaction between EGFR and annexin A1 after 10 min of EGF stimulation that was undetectable after 15 min stimulation (Figure S2E), consistent with annexin A1 tyrosine phosphorylation occurring prior to MCS formation.

Overall these data show that the requirements for MCS and EGF-stimulated ILV formation are similar, consistent with our hypothesis that annexin A1/S100A11 tether MCSs that provide sites for PTP1B-mediated effects on ILV formation.

### EGF-Stimulated ILV Formation Requires Cholesterol from Endocytosis of LDL or from Annexin A1-Dependent Transport from the ER

**Figure 3:**
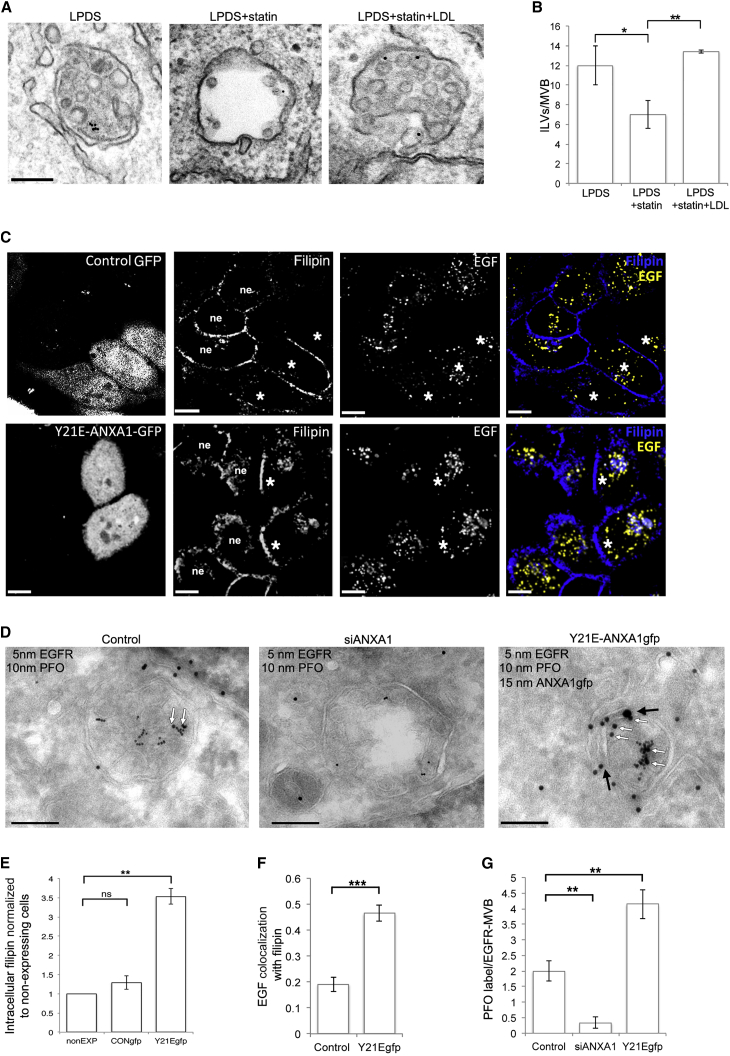
In the Absence of LDL, EGF-Stimulated ILV Formation Depends on Annexin A1-Dependent Transport of ER-Derived Cholesterol to EGFR-MVBs (A and B) HeLa cells cultured overnight in LPDS ± mevastatin (statin) ± LDL were stimulated with EGF and anti-EGFR gold for 30 min. Scale bar, 200 nm (A). ILVs/EGFR-MVB were quantified (B). Data are means ± SD of three experiments. ^∗^p < 0.05, ^∗∗^p < 0.01. (C, E, and F) HeLa cells transfected with control-GFP plasmid or phosphomimetic Y21E-ANXA1gfp were stimulated with fluorescent EGF for 30 min and filipin-stained for cholesterol. ne, non-expressing cells; ^∗^GFP-expressing cells. Scale bar, 5 μm (C). Intracellular filipin staining was measured in non-expressing cells (nonEXP) and cells expressing the control-GFP plasmid (CONgfp) or phosphomimetic annexin A1 (Y21Egfp) and normalized to non-expressing cells; data are means ± SD of three experiments (E). Colocalization (Mander’s coefficient) between EGF and filipin was determined in non-expressing control cells (Control) or cells expressing Y21E-annexin A1-gfp (F). Data are means ± SD of three experiments. ns, not significant p > 0.05; ^∗∗^p < 0.01, ^∗∗∗^p < 0.001. (D and G) HeLa cells transfected with Y21E-annexin A1-gfp or siRNA targeting annexin A1 (siANX) were cultured overnight in LPDS prior to stimulation with EGF and 5 nm-anti-EGFR-gold conjugate. Cryosections were labeled with PFO (10 nm) and GFP (15 nm). Black arrows indicate MCSs. White arrows show PFO label on ILVs. Scale bar, 200 nm (D). PFO gold/EGFR-MVB was quantified. Data are means ± SEM (G). ^∗∗^p < 0.01. See also Figure S3.

As MVBs mature, the number of ILVs increases. Since ILVs are rich in cholesterol (Mobius et al., 2003), we hypothesized that, just as plasma membrane cholesterol is required for endocytosis (Kozik et al., 2013), cholesterol-rich membrane may be required for ILV formation. The source of the cholesterol could be either exogenous sterols taken up via endocytosis of low-density lipoprotein (LDL) that is hydrolyzed in the endocytic pathway to release free cholesterol, or de novo synthesis in the ER. To test the cholesterol dependence of EGF-stimulated ILV formation, we removed both potential cholesterol sources by culturing cells in LPDS with or without statin to block cholesterol synthesis (Goldstein and Brown, 2015). The addition of statin reduced ILV numbers/MVB by over 40% (Figures 3A and 3B). Statins act early in the cholesterol biosynthesis pathway (Figure S3A), also affecting protein prenylation, and potentially therefore Rab function. However, the effect of statins on ILV formation was not due to reduced protein prenylation, because addition of LDL (Figures 3A and 3B) or the cholesterol precursor squalene (Figures S3A and 3B) reversed the effects of statin treatment on ILV formation. These results demonstrate the cholesterol dependence of EGF-stimulated ILV formation, which, in the absence of endocytosed LDL-cholesterol, is derived from the ER.

Increasing the need for sterol traffic from ER to EGFR-MVBs, by culturing cells in LPDS, increased the numbers of contacts between the two organelles (Figure 1B). This led us to ask if further increasing contacts between ER and EGFR-MVBs leads to a discernible increase in sterol traffic from the ER to EGFR-MVBs. We therefore examined whether overexpression of phosphomimetic annexin A1, which increases ER contacts with EGFR-MVBs (Figure 2F), also leads to an increase in free endosomal cholesterol in cells cultured in LPDS. Overexpression of Y21E-annexin A1-gfp increased intracellular filipin staining compared with gfp-transfected controls (Figure 3C, quantitated in E), which overlapped with endocytosed EGF (Figure 3C, quantitated in F). This colocalization was incomplete and predominantly with the population of EGF-positive endosomes in perinuclear clusters (Figures 3C and S3C). However, as filipin staining does not give a discrete signal, to demonstrate the presence of cholesterol in EGFR-MVBs we also used perfringolysin-O (PFO) to label cholesterol (Kwiatkowska et al., 2014). PFO is a well-characterized cholesterol-binding toxin (Iwamoto et al., 1997) that has been extensively used for quantitative cholesterol analysis (Das et al., 2014, Sokolov and Radhakrishnan, 2010) with increased PFO staining in the endocytic organelles of NPC1-deficient cells reported in several studies (for example, Kwiatkowska et al., 2014, Sugii et al., 2003). The PFO fused with glutathione S-transferase used in this study showed selective and dose-dependent recognition of cholesterol (Kwiatkowska et al., 2014), and we have confirmed its suitability as a quantitative cholesterol probe by cryo-immunoEM (see Experimental Procedures and Figures S3D and S3E). Supporting our observations with filipin staining, PFO immunogold labeling of cryosections of cells cultured in LPDS revealed an approximately 2-fold increase in PFO staining/EGFR-MVB in Y21E-annexin A1gfp-expressing cells (Figures 3D and 3G) that have increased MCSs (Figure 2F) compared with non-expressing controls. To further probe the role of annexin A1-regulated MCSs in cholesterol transport under these conditions, we quantified PFO staining in cells depleted of annexin A1 and cultured in LPDS. Loss of annexin A1 caused a >4-fold reduction in PFO staining/EGFR-MVBs (Figures 3D, 3G, and S3F), further supporting a role for annexin A1-regulated MCSs in ER to EGFR-MVB cholesterol transport.

### EGF-Stimulated ILV Formation in the Absence of LDL Requires VAP-ORP1L Interaction

**Figure 4:**
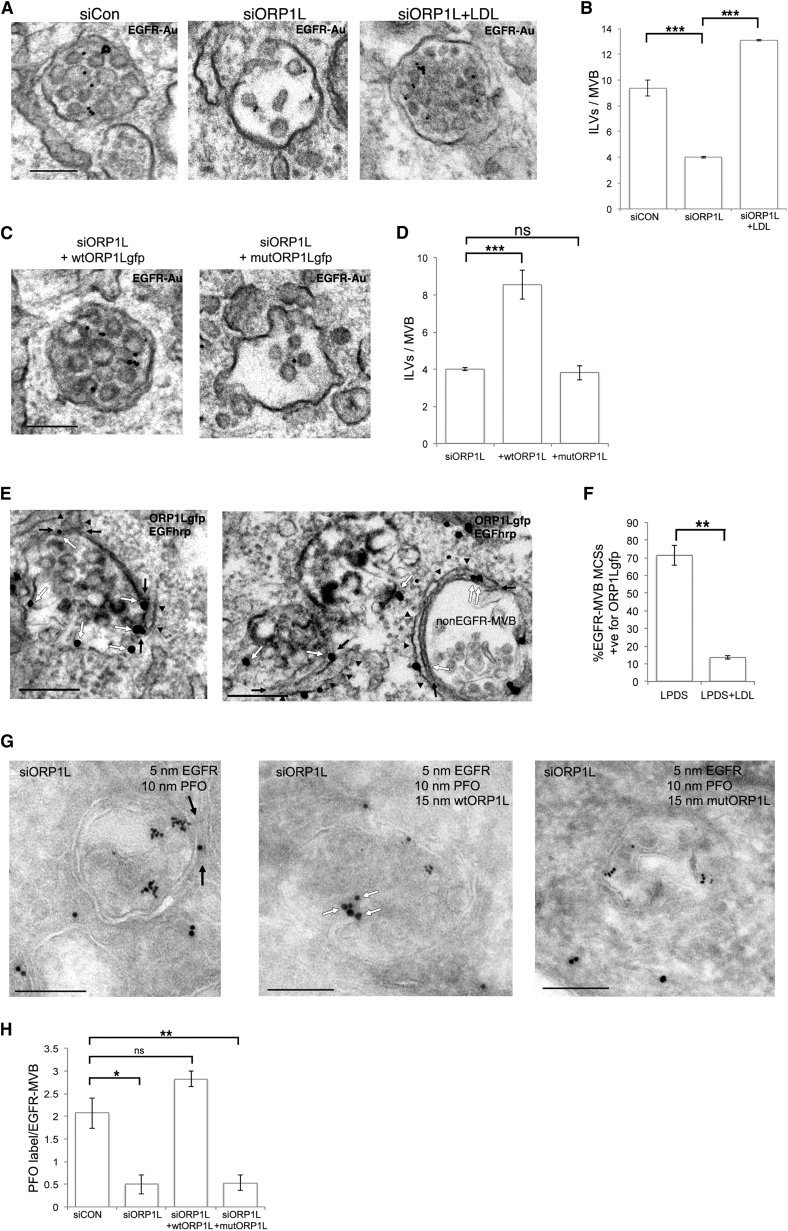
In the Absence of LDL, EGF-Stimulated ILV Formation Depends on Interaction between VAPs and ORP1L (A and B) HeLa cells transfected with a non-targeting siRNA (siCON) or siRNA targeting ORP1L (siORP1L) were cultured overnight in LPDS ± LDL and stimulated with EGF and anti-EGFR gold. Scale bar, 200 nm (A). The number of ILVs/EGFR-MVB was quantified and data shown as mean ± SD of three experiments (B). ^∗∗∗^p < 0.001. (C and D) HeLa cells depleted of ORP1L (siORP1L) were transfected with wtORP1L-gfp or an FFAT motif mutant ORP1L-gfp construct (mutORP1L-gfp), cultured overnight in LPDS and stimulated with EGF and anti-EGFR gold (EGFR-Au). Scale bar, 200 nm (C). The number of ILVs/EGFR-MVB was quantified and data are shown as means ± SD of three experiments (D). ns, not significant p > 0.05; ^∗∗∗^p < 0.001. (E and F) HeLa cells transfected with ORP1L-gfp were cultured overnight in LPDS ± LDL, stimulated with EGF-HRP (30 min) and stained for GFP using pre-embedding labeling (E). ORP1L-gfp staining (white arrows) was observed on EGFR-MVBs (containing electron-dense EGF-HRP/DAB reaction product) and at MCSs (black arrows) with the ER (arrowheads) and was associated with extended MCSs in non-EGFR-MVBs. Scale bar, 200 nm (E). ORP1L labeling at EGFR-MVB MCSs ± LDL was quantified (F). Data are means ± SD. ^∗∗^p < 0.01. (G and H) HeLa cells transfected with non-targeting siRNA (siCON) or siRNA targeting ORP1L (siORP1L) were transfected with wtORP1L-gfp or FFAT motif mutant ORP1L-gfp (mutORP1L) and cultured overnight in LPDS prior to stimulation with EGF and 5 nm-anti-EGFR-gold conjugate. Cryosections were labeled with PFO (10 nm) and GFP (15 nm). Black arrows show MCSs. White arrows show PFO label within EGFR-MVBs. Scale bar, 200 nm (G). PFO gold particles/EGFR-MVB were quantified. Data are means ± SEM (H). ns, not significant p > 0.05; ^∗^p < 0.05, ^∗∗^p < 0.01. See also Figure S4.

Having established a role for annexin A1-dependent transport of cholesterol from the ER to EGFR-MVBs in ILV formation, we next investigated candidate proteins that could play a role in cholesterol sensing and/or transfer at the contact. Our observation that ORP1L depletion, like LDL removal, resulted in increased MCSs specifically with EGFR-MVBs (Figure 1B) suggested a role for ORP1L in regulating endosomal sterol levels. This was confirmed by depletion of ORP1L, which reduced ILV formation in EGFR-MVBs by 50% in cells cultured in LPDS, an effect that was reversed by addition of LDL to the medium (Figures 4A and 4B). Since endosomal ORP1L interacts with ER-localized VAPA under conditions of low LDL-cholesterol, might this interaction promote cholesterol transfer from the ER to cholesterol-depleted EGFR-MVBs at MCSs in a manner analogous to the VAPA-OSBP-mediated ER to Golgi lipid transport (Mesmin et al., 2013)? Indeed, the 50% reduction of ILVs induced by ORP1L depletion was reproduced by depletion of VAPs (Figures S4A and S4B). This indicates that VAPs and ORP1L are required for transport of ER-derived cholesterol to support EGF-stimulated ILV formation. To further investigate the importance of the VAP-ORP1L interaction in ILV formation, we expressed WT or an FFAT motif mutant ORP1L construct (mutORP1L-gfp) that cannot bind VAP (Figure S4C) in cells depleted of ORP1L (Figures S4D–S4F). While WTORP1L expression was sufficient to reverse the effect of ORP1L depletion on EGF-stimulated ILV formation, a similar expression level of mutORP1L (Figures S4F and S4G) failed to correct the ILV phenotype (Figures 4C and 4D). Consistent with a role for ORP1L in the transport of ER-derived cholesterol to MVBs that is mediated by annexin A1-dependent MCSs, we found that the increased intracellular filipin staining observed on overexpression of Y21E-annexin A1 is blocked by ORP1L depletion (Figures S4H and S4I). ORP1L is recruited to MVBs by the late endosome marker, Rab7. How, therefore, can its role in EGF-stimulated ILV formation be reconciled with our previous observation that the ER forms MCSs with early EGFR-MVBs where ILV formation begins (Eden et al., 2010)? To determine whether ORP1L can be detected on EGFR-MVBs we expressed ORP1L-gfp in cells cultured with LPDS and found that ORP1L overexpression induced extended ER-MVB contacts (Figure S4J), but these were primarily with non-EGFR-MVBs (Figure 4E). Importantly, ORP1L was also present on EGFR-MVBs (Figure 4E) in the presence or absence of LDL, but only enriched at ER MCSs with EGFR-MVBs when cultured in the absence of LDL (Figure 4F). These results demonstrate a role for the ORP1L-VAP interaction in EGF-stimulated ILV formation. To determine if this interaction functions in sterol transfer to EGFR-MVBs under conditions of low LDL-cholesterol, we again expressed the FFAT motif mutant ORP1L construct in cells depleted of endogenous ORP1L. Consistent with a role for ORP1L in cholesterol transport to EGFR-MVBs under conditions of low endosomal cholesterol, depletion of ORP1L resulted in an approximately 4-fold reduction in PFO staining of cholesterol in EGFR-MVBs (Figures 4G and 4H). However, whereas expression of wtORP1L-gfp restored PFO labeling to levels comparable with control cells, mutant ORP1L expression had no effect on cholesterol label in EGFR-MVBs (Figures 4G and 4H). These data demonstrate the importance of the VAP-ORP1L interaction at ER-MVB contact sites for the transport of ER-derived cholesterol to EGFR-MVBs when cells are cultured in the absence of exogenous LDL.

## Discussion

Using EM, which allows unparalleled insights into MCS biology, we have identified multiple differentially regulated MCSs between the ER and endocytic organelles. We have demonstrated a role for VAPs in the regulation of MCSs with lysosomes and EGFR-negative MVBs. Surprisingly, neither VAPs nor ORP1L are required for ER MCSs with EGFR-MVBs in the presence of LDL-cholesterol. Instead these are a biochemically distinct subpopulation of contacts within the endocytic pathway that are specifically tethered by annexin A1 and its Ca^2+^-dependent ligand, S100A11. In vitro studies have shown that a heterotetramer of annexin A1 and S100A11 can bridge membranes alone (Gerke and Moss, 2002), and both proteins localize to ER contacts with EGFR-MVBs. How these proteins localize specifically to this subset of contacts is unclear but annexin A1 has been shown to associate with EGFR (Radke et al., 2004), and we identified an annexin A1-EGFR association that occurs rapidly after EGF stimulation and is relatively short-lived. The timing of this association suggests that it does not have a direct role in MCS formation, because the role of annexin A1 in MCS formation persists when the annexin A1-EGFR interaction is no longer detectable, but it may promote annexin A1 phosphorylation. Why tyrosine phosphorylation is so important for annexin A1's role in MCS and ILV formation is unclear. Whereas the endosomal association of the closely related annexinA2 requires its tyrosine phosphorylation (Morel and Gruenberg, 2009), endosomal association of annexin A1 appears more dependent on Ca^2+^-binding sites than N-terminal phosphorylation (Futter et al., 1993, Radke et al., 2004, Rescher et al., 2000). A combination of in vitro and in vivo experiments have suggested that phosphorylation of annexin A1 Y21 regulates not only Ca^2+^ sensitivity of endosome association (Futter et al., 1993), but also N-terminal proteolysis (Haigler et al., 1987) and sumoylation in response to EGF (Hirata et al., 2010). Together these could regulate protein-protein interactions and, thereby, MCS formation and stability. Interestingly expression of phosphomimetic Y21E-annexin A1 caused the greatest increase in MCS formation, but was less effective than WT annexin A1 at promoting ILV formation. Dephosphorylation of annexin A1 may have a role in MCS disassembly, which may be important to allow ILVs to detach from the MVB-limiting membrane. Although the dependence on EGF-stimulated phosphorylation suggests a specific requirement for annexin A1 in MCSs between the ER and EGFR-MVBs, a role for the closely related endosome-localized annexin A2 in other classes of MCS between the ER and endocytic pathway cannot be excluded.

That annexin A1-regulated MCSs allow PTP1B to interact with endosomal targets, including ESCRT0 (Eden et al., 2010, Stuible et al., 2010), provides a likely mechanism underlying annexin A1's role in ILV formation and emphasizes the importance of resolving the functional significance of ESCRT phosphorylation/dephosphorylation. Importantly, we find that annexin A1-regulated MCSs perform a dual role in ILV formation. Not only are they platforms for PTP1B-mediated effects at the endosome, but they also are required for the transport of ER-derived cholesterol to MVBs when endosomal cholesterol is low. A key finding of this study is that cholesterol is required for EGF-stimulated ILV formation. Interestingly, the ESCRT0 component, Hrs, which promotes ILV formation, has also been implicated in sterol sorting, and a role for Hrs in sorting cholesterol onto microdomains on the limiting membrane was proposed (Du et al., 2012). ESCRTII complexes have been shown to induce phase-separated microdomains that depend on cholesterol (Boura et al., 2012). Cholesterol-rich microdomains might initiate inward budding into the lumen of the MVB as the ILV begins to form, as has been found at the yeast vacuole (Wang et al., 2014).

ER MCSs have been implicated in specific lipid transfer steps at the Golgi, plasma membrane, and mitochondria (Lahiri et al., 2015). Transport of LDL-cholesterol from the endosome to the ER at MCSs has previously been proposed (Du et al., 2011, Raiborg et al., 2015b, van der Kant and Neefjes, 2014). In contrast, ER-endosome MCSs have never before been implicated in the transport of ER-derived cholesterol in the reverse direction, from the ER to endosomes. Here we demonstrate transport of ER-derived cholesterol to MVBs to support EGF-stimulated ILV formation, which requires the ORP1L-VAP interaction at MCSs when endosomal cholesterol is low. The Rab7-dependence of ORP1L recruitment means that it is primarily recruited to late endosomes and lysosomes. Annexin A1-MCSs form with both early and late endosomes that are defined not by their maturity but by the presence of EGFR. A Rab5 to Rab7 switch accompanies MVB maturation (Rink et al., 2005) but, consistent with our demonstration of ORP1L recruitment to EGFR-MVBs, both Rab5- and Rab7-positive MVBs can contain EGFR. The recruitment of increasing levels of ORP1L during MVB maturation allows a fundamental part of the MVB maturation process, ILV formation, to complete, even under conditions of low LDL-cholesterol.

A recent study in yeast identified an ER-anchored sterol transfer protein that is able to transfer sterols in vitro and to promote ergosterol-enriched domains that invaginate into the vacuole (Murley et al., 2015). Our finding of a role for annexin A1-regulated MCSs in the transport of ER-derived cholesterol to MVBs is the first evidence that ER-endosome MCSs function in lipid transport in mammalian cells. This transport requires ORP1L interaction with VAPs, which only occurs in low-cholesterol conditions (Rocha et al., 2009). In the absence of either LDL-cholesterol or ORP1L, MCS formation with EGFR-MVBs is increased, indicating that when LDL-cholesterol is present it is sensed by ORP1L, which limits MCS formation. How MCS size is regulated and the role of ORP1L in this process are yet to be determined. It is worth noting that MCSs between EGFR-MVBs and the ER have to be dynamically regulated to allow for localized disassembly before ILVs pinch off from the limiting membrane (to prevent ER from entering the invaginating vesicle). In the absence of LDL-cholesterol ORP1L binds VAP, and this interaction promotes cholesterol transfer from the ER to MVBs to support ILV formation. How the ORP1L-VAP interaction promotes cholesterol transfer remains to be resolved but it could function similarly to the OSBP-mediated ER to Golgi sterol transfer that is coupled to reverse transport of PI(4)P from the Golgi to the ER (Mesmin et al., 2013). A similar sterol-phosphoinositide exchange could also occur at ER-endosome contacts, with a pool of endosomal PI(4)P recently identified (Hammond et al., 2014). While direct sterol transfer at the MCS is the most likely explanation for our data, ORP1L effects on endosomal positioning could affect indirect sterol transport (via the plasma membrane), as ORP1L-VAP interactions increase peripheral positioning of endosomes by disrupting their association with minus-end directed microtubule motors (Rocha et al., 2009).

Our identification of key regulators of ER-endosome MCSs will enable the role of contact sites in lipid exchange between the endocytic pathway and the ER to be further explored. Transport of ER-derived cholesterol to MVBs ensures that EGF-stimulated ILV formation proceeds to completion, even when LDL-cholesterol in the endocytic pathway is low, as found for example in cells from familial hypercholesterolemia patients carrying mutations that prevent LDL uptake (Soutar and Naoumova, 2007). The evolution of such a mechanism underscores the importance of ILV formation for the spatial regulation of receptor tyrosine kinase signaling.

## Experimental Procedures

### Cell Culture and Transfection

HeLa cells were cultured in DMEM/10% fetal bovine serum (Invitrogen). Cells were transfected using Lipofectamine LTX Plus for plasmids and Lipofectamine RNAiMax (Invitrogen) for small interfering RNAs (siRNAs), according to the manufacturer's instructions.

### Antibodies, Plasmids, and siRNAs

A complete description is provided in the Supplemental Experimental Procedures.

### Electron Microscopy

Conventional and cryo-immunoEM were performed essentially as described (Eden et al., 2010) but with modifications detailed in Supplemental Experimental Procedures. EGFR-MVBs were distinguished from non-EGFR-MVBs by anti-EGFR gold particles and lysosomes were identified by multilamellar whorls. Apposing membranes at ER-MVB MCSs were <30 nm apart, with no minimum MCS length. MCS quantitation in random sections used methods validated by serial sectioning (Figure S1A), with >200 endocytic organelles/condition imaged over three experiments. For quantitation of ILVs or MCSs/EGFR-MVB, ≥100 EGFR-MVBs per condition were imaged by EM over three experiments. For quantitation of PFO label, 10 nm PFO gold was counted in EGFR-MVBs and SEM calculated in Microsoft Excel. For quantitation of ORP1L label at EGFR-MVB MCSs, cells were stimulated with EGF-horseradish peroxidase before fixation and 3,3-diaminobenzidine reaction. The number of ER MCSs with EGFR-MVB that are positive for ORP1L-gfp were counted in cells cultured overnight in LPDS ± LDL. For further details see Supplemental Experimental Procedures.

### Florescence Imaging

Immunofluorescence was essentially as previously described (Eden et al., 2010) with modifications as described in Supplemental Experimental Procedures.

### Western Blotting, Immunoprecipitation, and Quantitative RT-PCR

Described in full in Supplemental Experimental Procedures.

## Author Contributions

E.E., E.S.H., and A.T. conducted experiments, E.E., A.S., T.L., and C.F. designed experiments, and E.E., T.L., and C.F. wrote the paper.

## Supplemental Information

Document S1. Supplemental Experimental Procedures and Figures S1–S4

Document S2. Article plus Supplemental Information
