# Supplementary material for: Annexin A1 Tethers Membrane Contact Sites that Mediate ER to Endosome Cholesterol Transport
Source: Dev Cell. 2016 Jun 6;37(5):473–83. doi: 10.1016/j.devcel.2016.05.005 (PMC4906250; doi:10.1016/j.devcel.2016.05.005)
Supplement: Document S1. Supplemental Experimental Procedures and Figures S1–S4 [file mmc1.pdf]

Developmental Cell, Volume *37*

Supplemental Information

Annexin A1 Tethers Membrane Contact Sites that Mediate ER to Endosome Cholesterol Transport

Emily R. Eden, Elena Sanchez-Heras, Anna Tsapara, Andrzej Sobota, Tim P. Levine, and Clare E. Futter

SUPPLEMENTARY FIGURES

FIGURE S1

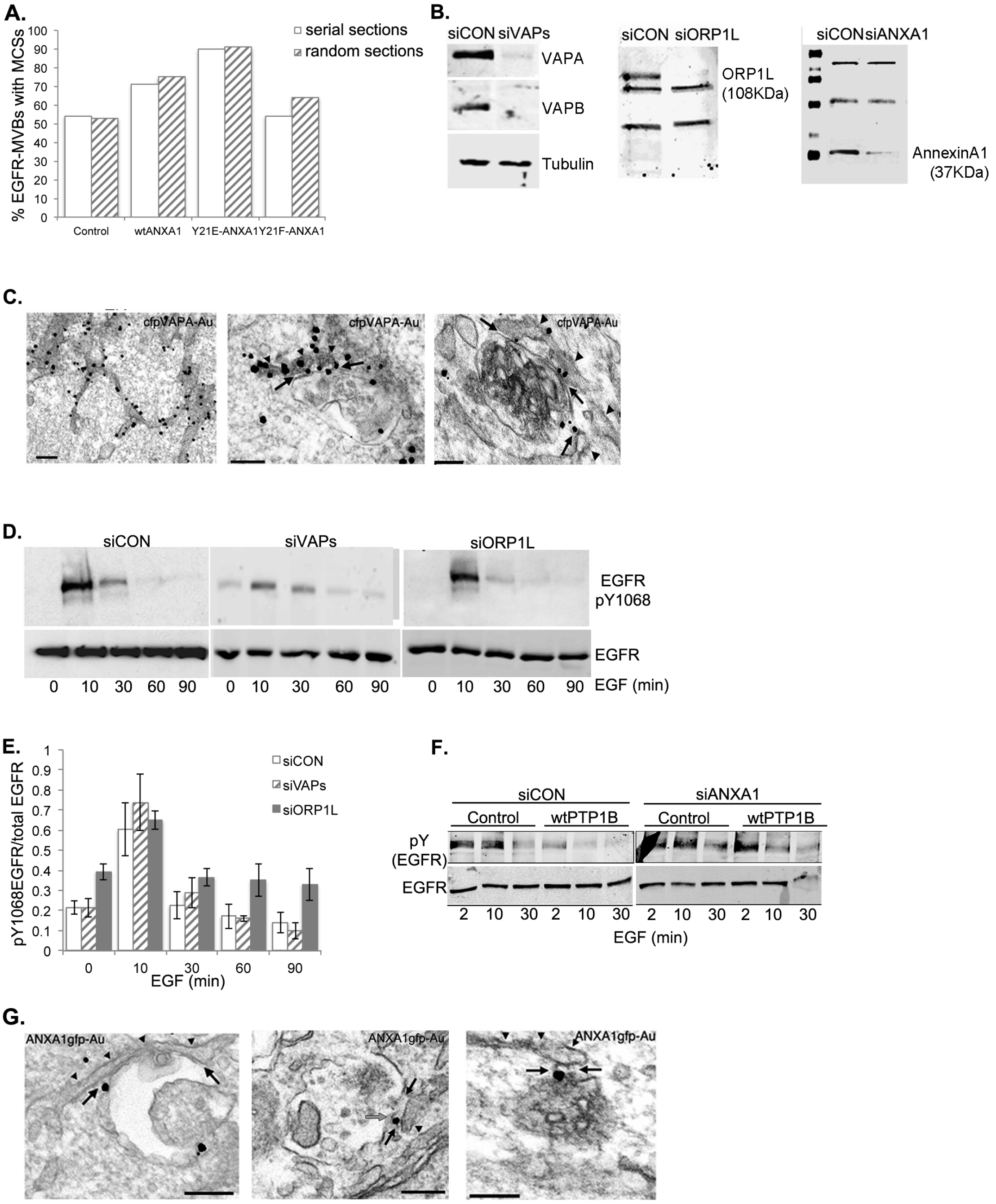

FIGURE S2

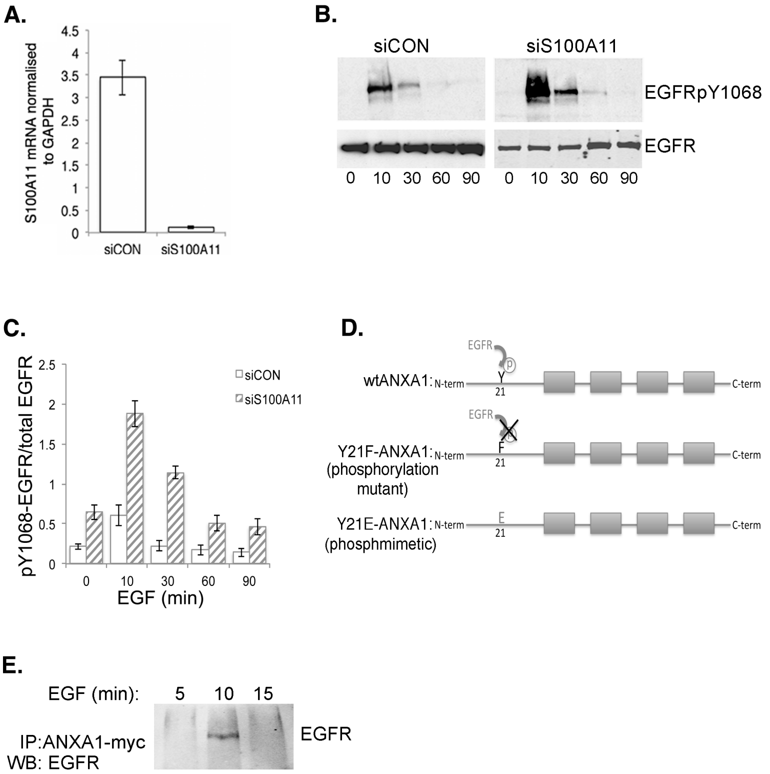
A.
S100A11 mRNA normalised to GAPDH
4.5
4
3.5
3
2.5
2
1.5
1
0.5
0
siCON
siS100A11
B.
siCON
siS100A11
EGFRpY1068
EGFR
0 10 30 60 90 0 10 30 60 90
C.
pY1068-EGFR/total EGFR
2.5
2
1.5
1
0.5
0
siCON
siS100A11
0 10 30 60 90
EGF (min)
D.
wtANXA1:
Y21F-ANXA1:
(phosphorylation mutant)
Y21E-ANXA1:
(phosphmimetic)
EGFR
N-term
C-term
Y
F
E
21
E.
EGF (min): 5 10 15
IP:ANXA1-myc
WB: EGFR
EGFR

FIGURE S3

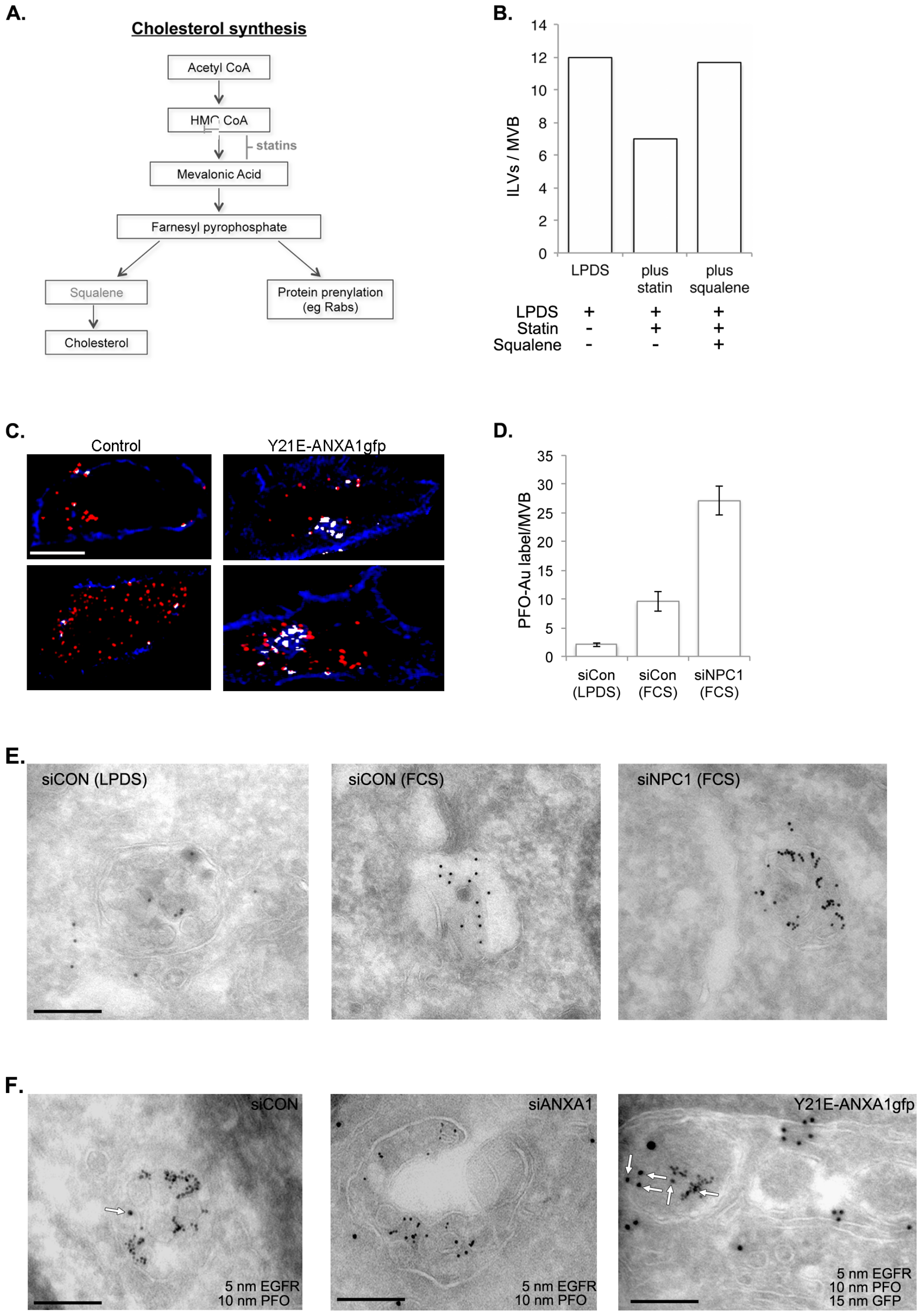
A.
Cholesterol synthesis
Acetyl CoA
HMG CoA
statins
Mevalonic Acid
Farnesyl pyrophosphate
Squalene
Protein prenylation (eg Rabs)
Cholesterol
B.
ILVs / MVB
LPDS
plus statin
plus squalene
LPDS + + +
Statin - + +
Squalene - - +
C.
Control
Y21E-ANXA1gfp
D.
PFO-Au label/MVB
siCon (LPDS)
siCon (FCS)
siNPC1 (FCS)
E.
siCON (LPDS)
siCON (FCS)
siNPC1 (FCS)
F.
siCON
siANXA1
Y21E-ANXA1gfp
5 nm EGFR
10 nm PFO
15 nm GFP

FIGURE S4

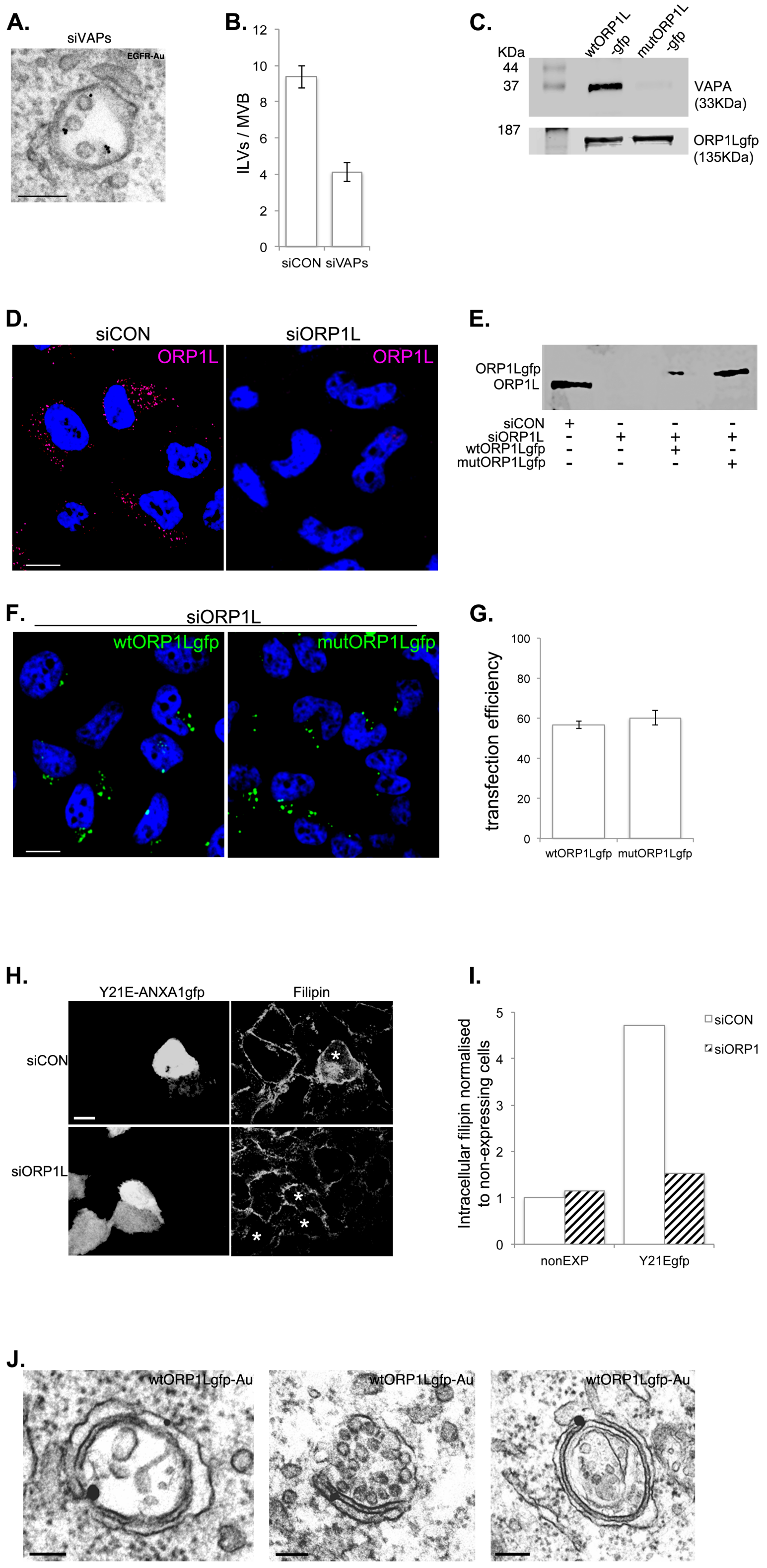

A.
siVAPs
EGFR-Au
B.
ILVs / MVB
siCON siVAPs
C.
wtORP1L -gfp
mutORP1L -gfp
KDa
44
37
187
VAPA (33KDa)
ORP1Lgfp (135KDa)
D.
siCON
siORP1L
ORP1L
E.
ORP1Lgfp
ORP1L
siCON
siORP1L
wtORP1Lgfp
mutORP1Lgfp
F.
siORP1L
wtORP1Lgfp
mutORP1Lgfp
G.
transfection efficiency
wtORP1Lgfp mutORP1Lgfp
H.
Y21E-ANXA1gfp
Filipin
siCON
siORP1L
I.
Intracellular filipin normalised to non-expressing cells
siCON
siORP1
nonEXP
Y21Egfp
J.
wtORP1Lgfp-Au

SUPPLEMENTARY FIGURE LEGENDS

Figure S1: Role of VAPs, ORP1L and annexin A1 in MCSs between the ER and MVBs (related to Figure 1).

(A) Hela cells mock transfected (Control) or transfected with wt-annexinA1 or annexinA1 mutants were stimulated with EGF and anti-EGFR 10nm gold and prepared for EM as described (Eden et al., 2010). MCSs with EGFR-MVBs were quantified in serial sections, with each MVB examined in every 70 nm section in which it was present (average of 6 sections/MVB). In parallel, cells were prepared for EM as described in supplemental experimental procedures and MCSs with EGFR-MVBs quantified in 70nm random sections

(B) Hela cells were transfected with non-targeting siRNA (siCON) or siRNA targeting VAPA and VAPB (siVAPs), ORP1L (siORP1L) or annexinA1 (siANXA1). Cell extracts were immunoblotted with antibodies for VAPA, VAPB, ORP1L, annexinA1, or tubulin, as indicated.

(C) Hela cells transfected with cfp-VAPA were fixed and stained for GFP using pre-embedding labelling with nanogold antibodies and gold enhancement prior to preparation for EM. Cfp-VAPA specifically labels the ER including sites of contact with endocytic organelles (arrows). Bar, 200nm.

(D,E) Hela cells transfected with non-targeting siRNA (siCON) or siRNA targeting VAPA and VAPB (siVAPs) or ORP1L (siORP1L) were stimulated with EGF for the indicated times, cell lysates blotted with antibodies to phosphotyrosine(pY)1068 or total EGFR (D), and quantified (E). Data are mean ± SD of 3 experiments.

(F) Hela cells stably overexpressing wtPTP1B or nontransfected controls were transfected with non-targeting siRNA (siCON) or siRNA targeting annexinA1 (siANXA1) and stimulated with EGF for the indicated times and treated as in (D).

(G) Hela cells transfected with annexinA1-gfp (ANXA1-gfp) were stained for gfp by pre-embedding labelling. Arrows indicate the position of MCSs between MVBs and the ER, Bar, 200nm.

Figure S2: Correlation between requirements for ER MCSs with EGFR-MVBs and intralumenal vesicle formation (related to Figure 2).

(A) RNA was isolated from Hela cells transfected with a non-targeting siRNA (siCON) or siRNA targeting S100A11 (siS100A11) for quantitative RT-PCR analysis of S100A11 expression. Values were normalised to GAPDH mRNA and data shown as mean ± SD of 3 experiments.

(B,C) Hela cells transfected with non-targeting siRNA (siCON) or siRNA targeting S100A11 (siS100A11) were stimulated with EGF for the indicated times, cell lysates blotted with antibodies to phosphotyrosine(pY)1068 or total EGFR (B), and quantified (C). Data are mean ± SD of 3 experiments.

(D) AnnexinA1 (wtANXA1) is phosphorylated at tyrosine residue (Y) 21 in the N terminus by EGFR kinase activity. Mutagenesis of Y21 results in annexinA1 that cannot be phosphorylated by EGFR kinase activity (phosphorylation mutant Y21F-ANXA1) or that mimics constitutive phosphorylation (phosphomimetic Y21E-ANXA1).

(E) Hela cells transfected with annexinA1-myc were stimulated with EGF for the indicated times and annexinA1-myc immunoprecipitates were western blotted with anti-EGFR antibody.

Figure S3: In the absence of LDL EGF-stimulated ILV formation depends on annexinA1-dependent transport of ER-derived cholesterol to EGFR-MVBs (related to Figure 3).

(A) Statins block the production of proteins required for prenylation as well as cholesterol. Squalene is a cholesterol precursor downstream of statin activity.

(B) Hela cells cultured overnight in delipidated serum in the absence (LPDS) or presence (plus STATIN) or statin and squalene (plus SQUALENE) were stimulated with EGF in the presence of anti-EGFR 10nm gold conjugate and prepared for EM. The number of ILVs per EGFR-MVB was quantified.

(C) Hela cells transfected with phosphomimetic Y21E-ANXA1gfp plasmid were cultured overnight in LPDS and stimulated with fluorescent EGF for 30 min. Co-localisation maps show that more intracellular filipin staining (blue) co-localises (white) with EGF (red) in Y21E-ANXA1gfp-expressing than in non-expressing (Control) cells. Scale bar, 5μm.

(D,E) Hela cells were transfected with non-targeting siRNA (siCON) or siRNA targeting NPC1 (siNPC1) and cultured overnight in medium containing 10% LPDS or 10% FCS. Cryosections were labelled for free cholesterol with PFO (10nm) and PFO label in MVBs quantified (D). Data shown are mean ± SEM. Representative images shown in E. The amount of PFO-labelling reflects the predicted endosomal cholesterol content (lowest in the absence of endocytosed cholesterol and highest when cholesterol egress from endosomes is blocked by NPC1 depletion). Scale bar, 200nm.

(F) Hela cells mock transfected (siCon) or transfected with siRNA targeting annexinA1 (siANXA1) or with Y21E-annexinA1-gfp plasmid (Y21E-ANXA1gfp) were cultured overnight in 10% LPDS prior to stimulation with EGF and 5nm-anti-EGFR-gold conjugate. Cryosections were labelled for free cholesterol with PFO (10nm) and for Y21E-annexinA1gfp (15nm). PFO label in EGFR-MVBs (white arrows) is absent in cells depleted of annexinA1, but increased in cells expressing Y21E-annexinA1gfp. Scale bar, 200nm

Figure S4: In the absence of LDL EGF-stimulated ILV formation depends on interaction between VAPs and ORP1L (related to Figure 4).

(A,B) Hela cells transfected with a non-targeting siRNA (siCON) or siRNA targeting VAPA and VAPB (siVAP) were cultured overnight in LPDS and stimulated with EGF and anti-EGFR gold (A). The number of ILVs per EGFR-MVB was quantified and data shown is mean ± SD of 3 experiments (B).

(C) Hela cells were transfected with wtORP1L-gfp or a FFAT motif mutant ORP1L-gfp construct (mutORP1Lgfp) prior to overnight incubation in LPDS. Anti-gfp immunoprecipitates were blotted with anti-VAPA or anti-ORP1L antibodies.

(D) Hela cells transfected with non-targeting siRNA (siCON) or siRNA targeting ORP1L (siORP1L) were fixed and stained for ORP1L by immunofluorescence.

(E-G). Hela cells depleted of ORP1L were transfected with wtORP1L-gfp or a FFAT motif mutant ORP1L-gfp construct (mutORP1Lgfp). Cell lysates were Western blotted with anti-ORP1L antibody (E) or cells were fixed and imaged by confocal microscopy (F). Transfection efficiencies were calculated as % cells expressing gfp and expressed as mean ± SD of 3 experiments (G).

(H,I) Hela cells treated with non-targeting siRNA (siCON) or siRNA targeting ORP1L (siORP1L) and cultured in LPDS were transfected with phosphomimetic Y21E-annexinA1-gfp (Y21E-ANXA1gfp), stimulated with EGF for 30 min and filipin-stained for free cholesterol. *:GFP-expressing cells; scale bar, 5μm (H). Intracellular filipin staining was measured in non-expressing cells (nonEXP) and cells expressing the phosphomimetic annexinA1 (Y21Egfp) and normalised to non-expressing cells (I).
(J) Hela cells transfected with ORP1L-gfp were fixed and stained for GFP using pre-embedding labelling with nanogold antibodies and gold enhancement prior to preparation for EM. ORP1L overexpression induces the formation of extended MCSs between MVBs and the ER that stain for ORP1L. Scale bar, 200nm.

SUPPLEMENTARY EXPERIMENTAL PROCEDURES:

Antibodies, plasmids and siRNAs: Mouse anti-VAPA and VAPB antibodies were from R&D Systems, rabbit anti-ORP1L was a kind gift from Vesa Olkkonen (University of Helsinki), rabbit anti-GFP was from Invitrogen, mouse anti-annexinA1 was from Dianova, rabbit anti-pY1068-EGFR was from Cell Signaling and sheep anti-EGFR was from Fitzgerald, rabbit anti-S100A11 was from Proteintech Europe and rabbit anti-GST was from Abcam. Antibody to the extracellular domain of EGFR used for gold conjugation was isolated from the Mab 108 hybridoma (ATCC).
Plasmids: cfpVAPA (Wyles et al., 2002) was a kind gift from Neale Ridgway (Dalhousie University), AnnexinA1gfp and Y21 mutants were as previously described(White et al., 2006), PTP1Bmyc and D181A mutant were as previously described (Eden et al., 2010) and wtORP1Lgfp and FFAT motif mutant were a kind gift from Jacques Neefjes (Netherlands Cancer Institute).
siRNAs: The non-targeting control siRNA was Allstars negative control siRNA (Qiagen 1027281). siRNA duplexes targeting VAPA (M-021382-01), VAPB (M-017795-00), ORP1L (D-008350-19) and S100A11 (D-012138-03) were synthesized by Dharmacon. The siRNA targeting annexinA1 was as previously described(White et al., 2006), synthesized by Qiagen.
Electron microscopy: For conventional EM, cells on thermanox coverslips (NUNC) in DMEM/10%FBS or DMEM/10%LPDS with or without 10μM Mevastatin (Sigma), or 1mM Squalene (Sigma) or 25μg/ml LDL were serum starved for 1h prior to stimulation with either 100ng/ml EGF (Sigma) or EGF conjugated to HRP with or without 10nm anti-EGFR gold conjugate in DMEM/0.2% BSA. After fixation in 2% paraformaldehyde (PFA)/2% glutaraldehyde for 30 min, cells were post-fixed in 1% osmium tetroxide, 1.5% potassium ferricyanide, incubated in 1% uranyl acetate, dehydrated and embedded in TAAB-812 resin. For calcium chelation, cells were incubated in 100mM BAPTA-AM during serum-starvation and EGF-stimulation. For pre-embedding labelling, EGF-HRP was prepared by mixing 5 μg of streptavidin-HRP (Invitrogen) and 0.3 μg of biotinylated EGF (Invitrogen) in 300 μl for 1h. Cells were stimulated with EGF-HRP, fixed in 4% PFA, permeabilised in 40μg/ml digitonin (Calbiochem) and incubated with primary and nanogold-secondary (Nanoprobes) antibodies prior to fixation for EM in 2% PFA/2% glutaraldehyde. Cells stimulated with EGF-HRP were then incubated with 1.5mg/ml DAB (TAAB) in TRIS buffer supplemented with 0.02% H_2O_2 for 30 min, prior to gold enhancement (Nanoprobes),

according to manufacturer's instructions. Post-fixation and embedding were as for conventional EM. For cryo-immunoEM cells were serum starved for 1h prior to stimulation with EGF and 5nm anti-EGFR gold conjugate. Fixation, processing and labelling were performed essentially as described (Eden et al., 2010). Ultrathin cryo-sections were labelled with mouse anti-myc, rabbit anti-GFP, or 20μg/ml PFO-GST (Kwiatkowska et al., 2014) followed by rabbit anti-GST and proteinA-gold.

EM Quantitation: As MCSs with a given MVB may not be in the plane of a random section, MCSs were initially quantified in serial sections. However, as this is extremely time consuming and not practical for the scale of the experiments described in this study, staining and embedding was optimized (as above), which allowed MCSs in random sections to be counted, essentially reproducing the data from serial sections (Figure S1A). Random sections were then used throughout the study. For ILV and MCS quantitation, means, standard deviations (SD) and paired, 2-tailed students T-tests were calculated in excel, with values >0.05 represented as not significant (ns), * <0.05, ** <0.01 and *** <0.001.

For quantitation of PFO labeling, cryo-immunoEM cholesterol labeling with PFO was first validated by comparing the number of PFO-gold particles per MVB in control cells cultured in LPDS (low cholesterol) or in medium containing 10% FCS (steady state) of in cells depleted of NPC1 and cultured in medium containing 10% FCS (high cholesterol). Culturing cells in FCS compared with LPDS resulted in an approximately 4-fold increase in PFO label at MVBs, while an additional 3-fold increase was observed in cells depleted of NPC1 (Figure S3D,E), confirming the suitability of PFO as a quantitative cholesterol probe by cryo-immunoEM.

Florescence Imaging: Cells on glass coverslips were fixed with 4% PFA in PBS for 20 min, washed and mounted in Prolong Gold antifade reagent with or without (filipin-stained cells) 4,6-diamidino-2-phenylindole (DAPI; Invitrogen). For EGF imaging, cells were serum starved for 1h and incubated with 200ng/ml Alexa 647-conjugated EGF (Invitrogen) in serum-free medium for 25-30 min prior to fixation. For cholesterol staining, cells were fixed in 4% PFA and incubated in 0.1mg/ml Filipin (Sigma). Images were acquired with a Leica DM-IRE2 microscope and TCS SP2 AOBS confocal system with a 63×/1.4 numerical aperture oil-immersion objective (Leica). Images were processed with Adobe Photoshop CS2 software (Adobe Systems). For quantitation, integrated densities relative to area were measured using ImageJ64 and normalized to controls. For colocalisation, manders coefficient was calculated using FIJI. Transfection efficiencies were calculated as the % of DAPI-stained cells positive for gfp. SDs were calculated in excel from data acquired over 3 experiments.

Western blotting and immunoprecipitation: Cells were lysed in lysis buffer (40 mM HEPES, 80 mM NaCl, 10 mM EDTA, 10 mM EGTA, 1% Triton-X100, protease inhibitor cocktail (Calbiochem set I), phosphatase inhibitor cocktail (Calbiochem set II), lysates were fractioned by SDS–PAGE on 10% gels under reducing conditions and immunoblotted on nitrocellulose membranes. Following incubation with infrared-fluorophore-conjugated secondary antibodies, membranes were scanned in an Odyssey SA scanner (LI-COR Biosciences). Band densities were measured using FIJI and SDs were calculated in excel from data acquired over 3 experiments. For immunoprecipitation, MYC- or GFP-tagged proteins were immunoprecipitated from cell lysates by incubation with Protein G PLUS-Agarose (Santa Cruz) and anti-MYC or -GFP antibody prior to immunoblotting.

Quantitative RT-PCR: RNA was isolated using RNeasy (Qiagen) and treated with DNAse (Applied Biosystems), prior to quantitative RT-PCR using Power SYBR Green PCR master mix (Applied Biosystems), according to manufacturer's instructions. Values were normalized to GAPDH in each sample. The primer sequences detailed below were synthesized by MWG:
S100A11-F: 5′-CCTTGACCGCATGATGAAGA-3′
S100A11-R: 5′-CTAGGCCACCAATCAGATTAAGA-3′
GAPDH-F: 5′-GGAGTCAACGGATTTGGTCGTA-3′
GAPDH-R: 5- GGCAACAATATCCACTTTACCAGAGT-3′

SUPPLEMENTARY REFERENCES:

Eden, E.R., White, I.J., Tsapara, A., and Futter, C.E. (2010). Membrane contacts between endosomes and ER provide sites for PTP1B-epidermal growth factor receptor interaction. Nature cell biology *12*, 267-272.

Kwiatkowska, K., Marszalek-Sadowska, E., Traczyk, G., Koprowski, P., Musielak, M., Lugowska, A., Kulma, M., Grzelczyk, A., and Sobota, A. (2014). Visualization of cholesterol deposits in lysosomes of Niemann-Pick type C fibroblasts using recombinant perfringolysin O. Orphanet journal of rare diseases *9*, 64.

White, I.J., Bailey, L.M., Aghakhani, M.R., Moss, S.E., and Futter, C.E. (2006). EGF stimulates annexin 1-dependent inward vesiculation in a multivesicular endosome subpopulation. The EMBO journal *25*, 1-12.

Wyles, J.P., McMaster, C.R., and Ridgway, N.D. (2002). Vesicle-associated membrane protein-associated protein-A (VAP-A) interacts with the oxysterol-binding protein to modify export from the endoplasmic reticulum. The Journal of biological chemistry *277*, 29908-29918.
